# Irrationalism, Ontological Stratification, and Health Behaviour During Pandemics: A Four-Tier Framework of Behavioural Response

**DOI:** 10.1192/j.eurpsy.2026.11889

**Published:** 2026-07-31

**Authors:** C. Lazzari

**Affiliations:** Psychiatry, International Centre for Healthcare and Medical Education (ICHME), London, United Kingdom

## Abstract

**Introduction:**

The COVID-19 pandemic destabilised not only public health systems but also fundamental constructs of identity, reason, and reality. Traditional models grounded in rationality and epidemiological logic proved inadequate for capturing how individuals and collectives behave under existential threat. Ontological stratification, as proposed by Guerrero Fernandez (2020), posits that pandemics unfold across layered realities—biological, social, and experiential—necessitating a philosophical reappraisal of public health behaviour.

**Objectives:**

To examine how irrationalism and solipsism shape behavioural responses to pandemics, particularly in the face of public health guidance. The study aims to introduce the Quadruplex Helix framework as a conceptual model for understanding health-related decision-making across individual, relational, peer, and cultural domains.

**Methods:**

The study employed a philosophical–clinical methodology, integrating traditions from metaphysics, epistemology, and phenomenology. Definitions of irrationalism were reviewed across philosophical and linguistic sources, while clinical literature and behavioural data were analysed to trace pandemic-era epistemic shifts. A four-tier analytical framework—the Quadruplex Helix of Pandemic Health Ontology—was developed to map behavioural stratification.

**Results:**

Findings indicate that irrationalism during crises operates as an adaptive epistemic response rather than a deviation from logic. At the micro level, individuals rejected public health measures based on subjective certainty. At the meso level, couples consolidated shared beliefs, occasionally manifesting in folie à deux. Peer groups at the macro level reinforced identity through opposition to norms, while at the mega level, cultural narratives fostered denialism and exceptionalism. These behaviours were not reducible to information deficits but reflected deeper ontological tensions.

**Image 2:**
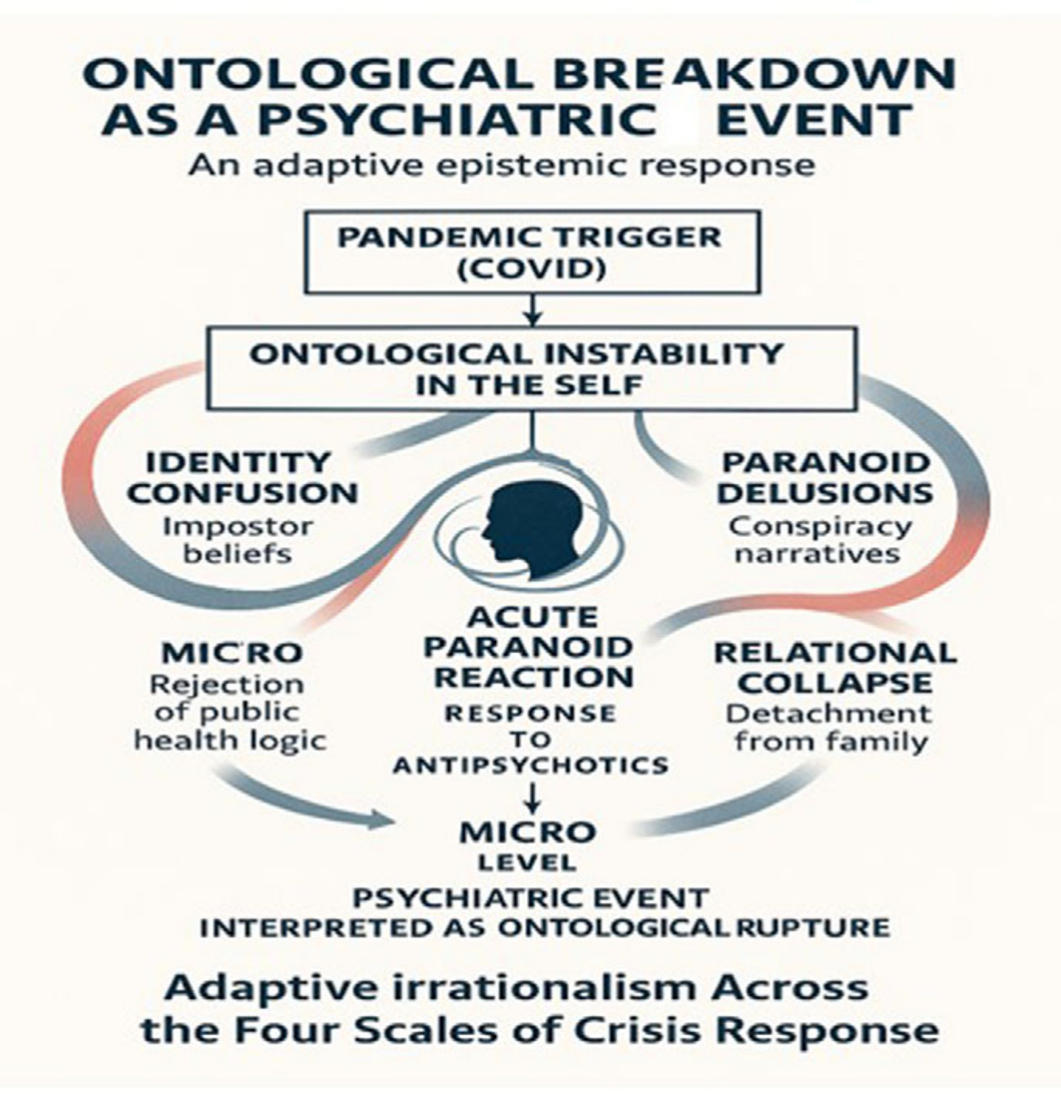
Image 2: Long description.

**Conclusions:**

Pandemic health behaviour is shaped by multi-layered ontologies wherein affect, belief, and identity override rationalist paradigms. The Quadruplex Helix provides a framework for understanding how metaphysical orientations and social embeddedness produce divergent responses to health crises. Recognising the philosophical dimensions of irrationalism and solipsism may inform more effective public health strategies and psychiatric engagement during future emergencies.

**Disclosure of Interest:**

None Declared

## Long descriptions

### Image 2: Long description

The diagram is titled ONTOLOGICAL BREAKDOWN AS A PSYCHIATRIC EVENT with the subtitle An adaptive epistemic response.

At the top, a box labeled PANDEMIC TRIGGER (COVID) has a downward arrow pointing to a second box labeled ONTOLOGICAL INSTABILITY IN THE SELF.

From this central instability, four curved, color-graded arrows (blue and red) lead to four distinct psychological states arranged in a circular layout around a central silhouette of a human head.

1. Top-Left: IDENTITY CONFUSION with the sub-text Impostor beliefs.

2. Top-Right: PARANOID DELUSIONS with the sub-text Conspiracy narratives.

3. Bottom-Left: MICRO Rejection of public health logic.

4. Bottom-Right: RELATIONAL COLLAPSE with the sub-text Detachment from family.

At the center of these four states is a circular icon containing a head silhouette, labeled ACUTE PARANOID REACTION and RESPONSE TO ANTIPSYCHOTICS.

A final downward arrow from this central core points to the base of the diagram, which reads: MICRO LEVEL PSYCHIATRIC EVENT INTERPRETED AS ONTOLOGICAL RUPTURE.

The footer text concludes: Adaptive irrationalism Across the Four Scales of Crisis Response.

Navigate back to Image 2:.

